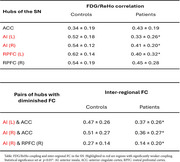# Connectivity‐Metabolism Interplay in the Salience Network: Insights into Network‐Specific Dysfunctions in Frontotemporal Dementia

**DOI:** 10.1002/alz70856_104964

**Published:** 2026-01-07

**Authors:** Mathew Joshy, Linshan Liu, Praveen Dassanayake, Marco Aiello, Udunna Anazodo, Elizabeth Finger, Keith St Lawrence

**Affiliations:** ^1^ University of Western Ontario, London, ON, Canada; ^2^ Lawson Research Institute, London, ON, Canada; ^3^ IRCCS SYNLAB SDN, Naples, Italy; ^4^ Montreal Neurological Institute, McGill University, Montreal, QC, Canada

## Abstract

**Background:**

The human brain is organized into macroscale functional networks that exhibit temporally synchronized spontaneous neural activity, known as functional connectivity (FC). Considering the high energetic cost of synaptic transmission (Tomasi et al. 2013, Aiello et al. 2015), hybrid imaging combining resting‐state functional MRI (rsfMRI) with ^18^F‐Fluorodeoxyglucose positron emission tomography (FDG‐PET) offers a unique opportunity to study the relationship between FC and energy demands. Alzheimer's is associated with significant dissociation between regional metabolism and neural activity, particularly within functionally active network hubs (Marchitelli et al. 2018); however, other neurodegenerative disorders remain unexplored. Frontotemporal dementia (FTD) is a rare form of dementia marked by functional breakdown of the salience network (SN), which regulates appropriate responses to stimuli. Like Alzheimer's, we hypothesized that FTD would be characterized by functional/metabolic dissociation; however, network‐level breakdown would be most evident in the SN given its role in the disease process.

**Method:**

FDG‐PET and rsfMRI were simultaneously collected on a Siemens Biograph mMR scanner from 18 controls and 20 behavioral‐variant FTD (bvFTD) patients. FDG maps were converted into standardized uptake value ratio (SUVr). Local FC was quantified as Regional Homogeneity (ReHo), an fMRI metric reflecting regional synchronization of neural activity. Voxel‐wise Spearman correlations were used to assess the relationship between ReHo and FDG‐SUVr. Furthermore, inter‐regional FC was measured with seed‐based FC analysis. Group comparisons were made using 2‐sample t‐tests (*p* <0.05) while correcting for multiple comparisons.

**Result:**

Reduced correlations between FDG and ReHo were found within the hubs of the SN in bvFTD, particularly bilateral anterior insula (AI) (Table). Analysis of inter‐regional FC revealed diminished communication between the AI and other SN hubs (Table).

**Conclusion:**

The disconnection between local FC and metabolism in the anterior insula (AI), coupled with disrupted intra‐network communication within the SN, supports the hypothesis of insula being a primary target in FTD (Seeley, 2010). These findings indicate a critical role of FC/metabolism coupling in maintaining network integrity and suggests that its disruption may lead to progressive breakdown of the SN, contributing to the functional deficits characteristic of the disease.